# Deep-learning based morphological segmentation of canine diffuse large B-cell lymphoma

**DOI:** 10.3389/fvets.2025.1656976

**Published:** 2025-08-25

**Authors:** Kenneth Ancheta, Androniki Psifidi, Andrew D. Yale, Sophie Le Calvez, Jonathan Williams

**Affiliations:** ^1^Pathobiology and Population Science, Royal Veterinary College, Hatfield, United Kingdom; ^2^Clinical Science and Services, Royal Veterinary College, Hatfield, United Kingdom; ^3^IDEXX Laboratories Ltd., Wetherby, United Kingdom

**Keywords:** deep learning, lymphoma, canine, digital pathology, artificial intelligence

## Abstract

Diffuse large B-cell lymphoma is the most common type of non-Hodgkin lymphoma (NHL) in humans, accounting for about 30–40% of NHL cases worldwide. Canine diffuse large B-cell lymphoma (cDLBCL) is the most common lymphoma subtype in dogs and demonstrates an aggressive biologic behaviour. For tissue biopsies, current confirmatory diagnostic approaches for enlarged lymph nodes rely on expert histopathological assessment, which is time-consuming and requires specialist expertise. Therefore, there is an urgent need to develop tools to support and improve veterinary diagnostic workflows. Advances in molecular and computational approaches have opened new avenues for morphological analysis. This study explores the use of convolutional neural networks (CNNs) to differentiate cDLBCL from non-neoplastic lymph nodes, specifically reactive lymphoid hyperplasia (RLH). Whole slide images (WSIs) of haematoxylin-eosin stained lymph node slides were digitised at 20 × magnification and pre-processed using a modified Aachen protocol. Extracted images were split at the patient level into training (60%), validation (30%), and testing (10%) datasets. Here, we introduce HawksheadNet, a novel lightweight CNN architecture for cancer image classification and highlight the critical role of stain normalisation in CNN training. Once fine-tuned, HawksheadNet demonstrated strong generalisation performance in differentiating cDLBCL from RLH, achieving an area under the receiver operating characteristic (AUROC) of up to 0.9691 using fine-tuned parameters on StainNet-normalised images, outperforming pre-trained CNNs such as EfficientNet (up to 0.9492), Inception (up to 0.9311), and MobileNet (up to 0.9498). Additionally, WSI segmentation was achieved by overlaying the tile-wise predictions onto the original slide, providing a visual representation of the diagnosis that closely aligned with pathologist interpretation. Overall, this study highlights the potential of CNNs in cancer image analysis, offering promising advancements for clinical pathology workflows, patient care, and prognostication.

## 1 Introduction

Canine diffuse large B-cell lymphoma (cDLBCL) is the most common subtype of lymphoma in dogs, typically arising in a multicentric form, and is characterised by an aggressive biological behaviour (1–3). The current gold standard treatment for cDLBCL is multi-agent maximum tolerated dose chemotherapy; the CHOP protocol (cyclophosphamide, doxorubicin, vincristine, and prednisolone) currently confers 1-, 2-, and 3-year survival rates of 20, 13, and 8%, respectively. Accurate diagnosis is important to inform effective therapeutic intervention, which directly impacts prognostic outcomes. While fine-needle aspirate cytological screening and biopsy analysis are commonly used for investigating enlarged lymph nodes, lymphoma diagnosis can be a challenging task for pathologists due to the similarity in appearance of neoplastic and normal lymphocytes and the complex classification of canine lymphomas (2). Therefore, there remains an urgent and unmet need to develop better and cost-effective tools to better diagnose and prognosticate cDLBCL patients.

A convolutional neural network (CNN) is a category of deep learning (DL) architecture that effectively detects important features without human supervision. One effective application of CNN models is computer vision tasks [reviewed in (4)], including the analysis of histological images for human (5–9) and veterinary sciences (10, 11). Different applications of CNN models have been developed for veterinary settings as reviewed in (12, 13). In human DLBCL (hDLBCL), Li et al. (14) developed a CNN platform that resulted in a near-perfect diagnostic accuracy across three different hospitals, outperforming experienced pathologists. Ferrandez et al. (15) developed a model that infers time-to-progression of hDLBCL patient from positron emission tomography images within 2 years compared to international guidelines (15). Recently, Lee et al. (16) and colleagues showcased a model that predicts hDLBCL prognosis in patients treated with immunochemotherapy (rituximab + CHOP). In the veterinary field, CNN models were developed to differentiate different types of canine lymphomas (10) and infer diet-versus steroid-based treatment response of dogs affected with protein-losing enteropathy (11). Although niche studies exist, there remains a need to develop better computer vision models for morphological analysis in underrepresented fields like veterinary science, which lags behind human pathology in adoption of such tools.

Training reliable and robust CNN models for morphological analysis can be computationally demanding, requiring effective image pre-processing and careful hyperparameter fine-tuning. Many pre-trained CNNs previously used to develop morphological models involve complex architectures, extensive fine-tuning (5–9), and often require high-performance computing (HPC) systems for training. Moreover, whole slide images (WSIs) are commonly used to train models for histological slide analysis. Although WSIs provide an abundant source of data for CNN training, they can be challenging to pre-process due to their large size and inconsistent staining. Image processing workflows, such as the Aachen protocol, offer a potential standardisation method for image pre-processing in DL, as demonstrated in various studies (9, 17–20). However, such workflows may not be optimal for all types of tissue, particularly regarding the stain normalisation step (21). There is a need to develop a more dynamic workflow that can be applied to different data types for image pre-processing, along with a lightweight CNN capable of generating reliable models for morphological analysis.

The primary purpose of this study is to determine whether CNN models can be trained to differentiate between cDLBCL and non-neoplastic canine lymph nodes. Additionally, this paper introduces a new lightweight CNN architecture, HawksheadNet, for training computer vision models. Finally, this paper highlights alternative methods for pre-processing lymph node slide images for DL applications.

## 2 Materials and methods

### 2.1 Patient cohort

The study cohort consisted of lymph node histopathology samples from 127 cases definitively diagnosed with either cDLBCL or reactive lymphoid hyperplasia (RLH), collected between 1st July 2021 and 31st December 2022. Haematoxylin-eosin (HE)-stained slides were collected from IDEXX Laboratories, United Kingdom. Of these, 59 were cDLBCL and 68 were RLH. Aside from malignancies, one of the key clinical differential diagnose of canine lymphadenopathy is RLH (10). RLH is characterised by non-neoplastic polyclonal lymphocytes that often resolves after antigen clearance (22). In contrary to RLH, cDLBCL consists of an uncontrollable monoclonal neoplastic expansion. Therefore, RLH was used as the contrasting control to cDLBCL to represent non-neoplastic lymphocyte proliferation. cDLBCL or RLH diagnosis was performed by board-certified anatomic pathologists (IDEXX) based on the combination of cellular morphology, immunohistochemistry (IHC), and/or PCR for antigen receptor rearrangements (PARR). For cDLBCL samples without follow-up IHC or PARR, the morphology of the neoplasm was additionally corroborated as large cell lymphoma consistent with presumptive cDLBCL by a board-certified pathologist (JW). For all cases used in model development and testing, the 12 patients with cDLBCL had results for CD79a and CD3 IHC, and one had CD3 plus CD20, while among the RLH cases, one had PARR testing to exclude neoplasia where morphology alone was not definitive. This study primarily focused on patients with enlarged peripheral lymph nodes; therefore, cases with only visceral lymph node involvement were excluded from downstream analysis.

### 2.2 Whole slide image scanning

HE-stained slides were digitised at 20 × magnification using a Zeiss AxioScan. Z1 Slide Scanner (Carl Zeiss Microscopy, Oberkochen, Germany). All whole slide images (WSIs) are exported in Carl Zeiss Image (CZI) file.

### 2.3 Whole slide image pre-processing for deep learning

Due to the data and pixel size of a typical WSI, it is challenging to use an entire WSI to take advantage of the entire scan for DL. For this study, we employed a modified Aachen Protocol to extract smaller tiles from WSIs for DL. The Aachen protocol was employed to pre-process WSI for training computer vision DL models for histopathology, as previously described (11, 17, 18, 20, 23). Based on this protocol, QuPath (v0.5.1 ×64) (24) was employed to tesselate WSIs into non-overlapping 512 × 512 pixels tiles at 0.22 μm per pixel resolution using an in-house groovy file (see Data and code statement). Regions of interests (ROIs) were selected in Qupath that included relevant lymph node tissue exhibiting lymphoid hyperplasia or lymphoid neoplasia with generated tiles occasionally also containing adjacent non-diagnostic tissue (e.g., adipose, blood vessels) (2, 25). Exclusion criteria included any non-lymphoid tissue encountered within WSIs such as muscle, mammary tissue, necrotic areas, haemorrhages, glands and other histological artefacts.

### 2.4 Haematoxylin-eosin stain normalisation

**Figure 1:**
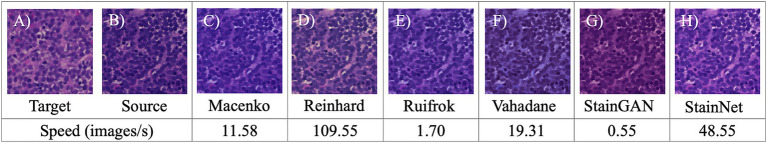
Visual comparison of different stain normalisation methods and normalisation speed. Visual comparison of different stain normalisations. **(A)** Target image used as references for stain transfer-based algorithms, **(B)** source or sample image being normalised, stain transfer-based normalisers **(C)** Macenko, **(D)** Reinhard, **(E)** Ruifrok, and **(F)** Vahadane as well as convolutional neural network (CNN)-based stain normalisers such as **(G)** StainNet and **(H)** StainGAN. The speed of the stain normalisation algorithm is shown on below the normalised images as image/s.

While histopathology laboratories usually apply a standardised HE-staining process, variable factors such as reagents, staining intensity, age of slides, and light exposure can alter staining quality and introduce inconsistencies (26). Statistics-based stain normalisation algorithms including Macenko, Reinhard, Ruifrok, and Vahadane were implemented using TIAToolbox (v1.5.1) (27), as well as a generative adversarial network (GAN)-based normaliser StainGAN (28) and StainNet (29) (Figure 1). The pre-trained models used for normalisation with StainGAN and StainNet were developed by Kang et al. (29), which were both trained on HE-stained human metastatic lymph node WSIs from breast cancer patients (CAMELYON16) to address stain inconsistencies across all samples. The speed of the stain normaliser algorithm was calculated based on the average number of images processed per second across three runs using 100 non-normalised images.

### 2.5 Data pre-processing for supervised machine learning

Patient overrepresentation was prevented by limiting the maximum number of tiles per case to the median tile count. For cases with more extracted tiles than the median value, a number of images equal to median were randomly selected, while all the tiles from cases with fewer tiles than the median were included. A patient-level data split was performed to ensure that tiles from a single case were not represented in more than one data set. Assigning adjacent tiles into training/validation set and another for testing can introduce data leakage. Data leakage can lead to overly optimistic results in CNN models, which is the process referred to as the incidence in which information is used during model training that is not expected to be available until testing (30). In this study, a patient-level data split ensured that all images from a single clinical case to only appear in one dataset. For example, if a patient was set in training set, their images were excluded from validation or testing set and vice versa. Patients were randomly assigned into training, validation and testing sets in a 60:30:10 ratio, respectively. To preserve the class ratios based on the original data, a stratified data split was applied using the Scikit-learn (v1.1.3) function StratifiedShuffleSplit(). Class information was retained for the supervised machine learning (SML) approach.

### 2.6 Deep learning model training, data properties, and hyperparameters

**Figure 2:**
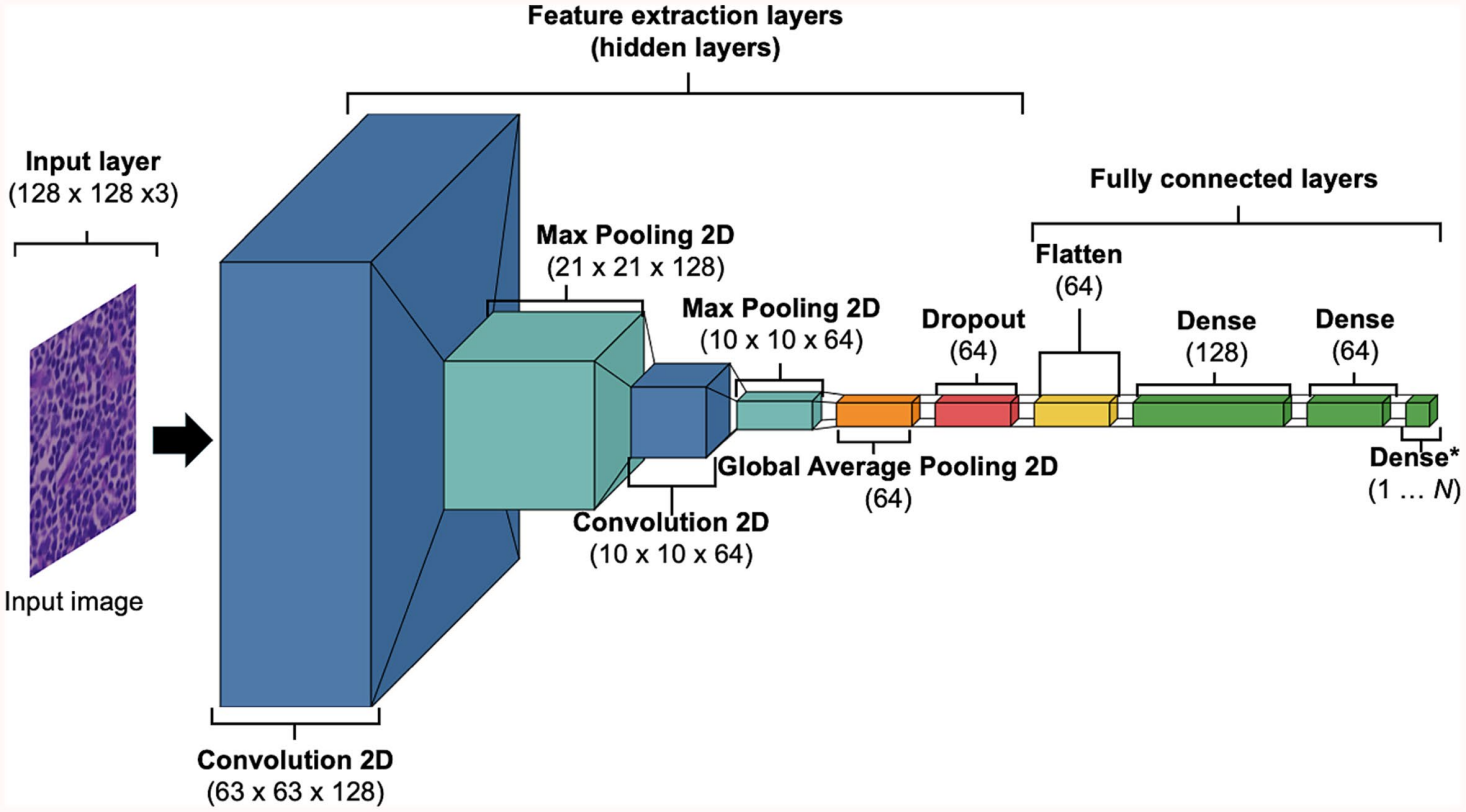
Baseline HawksheadNet architecture. The proposed custom CNN architecture referred to as HawksheadNet showcases a compact build. The baseline HawksheadNet has an input later of 128 × 128 × 3, followed by two sequential convolutional layers with decreasing filters starting with 128 to 64, each convolutional block is interspersed with maxpooling 2-dimentional layers to downsample the feature maps. A global average pooling layer is applied to reduce spatial dimensions, followed by a dropout layer with a 10% dropout rate for regularisation. Finally, the extracted features pass through three fully connected dense layers (128, 64, and 1 neuron, respectively), with the last layer employing a sigmoid activation function for binary classification.

DL models were developed using training and validation sets. The testing set was reserved until performance testing and was treated as ‘unseen’ or ‘never-seen’ data, representing real world data. The term ‘unseen’ or ‘never-seen’ indicates that the test data was not involved in model training or optimisations, but was subjected to the same preprocessing steps, including stain normalisation, as the training data prior to evaluation. For the supervise machine learning approach, the data were represented as $\left[X_{i}(x_{j},y_{i})|\;I=1\ldots N\right]$. Here, *N* indicates the total number of tiles, $X_{i}$ represents the WSI that was being tiled into smaller images $x_{j}$ which carries a label $y_{i}$ ∈ [0, 1] inherited from $X_{i}$ (e.g., 0 = RHL, 1 = cDLBCL). Training and testing performances were completed using TensorFlow (v2.10.0). Tiles $x_{j}$ were fed into our custom convolutional neural network (CNN), we referred to as HawksheadNet (Figure 2). HawksheadNet features a relatively lightweight architecture, starting with an input layer of 128 pixels in width and height, with three colour channels—Red, Green, and Blue (RGB)—resulting in dimensions of 128 × 128 × 3, followed by two convolutional blocks with progressively decreasing filters (from 128 to 64), interspersed with max-pooling layers. A global average pooling layer is subsequently applied, followed by dropout regularisation set to randomly drop 25% of neurons. The output is then passed through three consecutive dense layers, with the final layer used for classification (Figure 2). The hyperparameters were configured as follows: an initial learning rate of 0.0001, a batch size of 512, Adaptive Moment Estimation (AdAM) optimiser, a loss function based on binary cross-entropy, and models were trained for 100 epochs.

### 2.7 Model optimisation and fine-tuning

The number of tiles per class within each dataset were equalised by randomly removing tiles from the class with a higher number of images. See Supplementary method 1 for fine-tuning steps. Additionally, L1 and L2 regularisation penalties were applied to HawkheadNet to determine whether they can improve generalisation and limit overfitting. L1 regularisation reduces overfitting by shrinking some weights to zero, enabling feature selection, while L2 regularisation minimises overfitting by shrinking weights to small values without eliminating them (31).

### 2.8 Transfer learning

**Figure 3:**
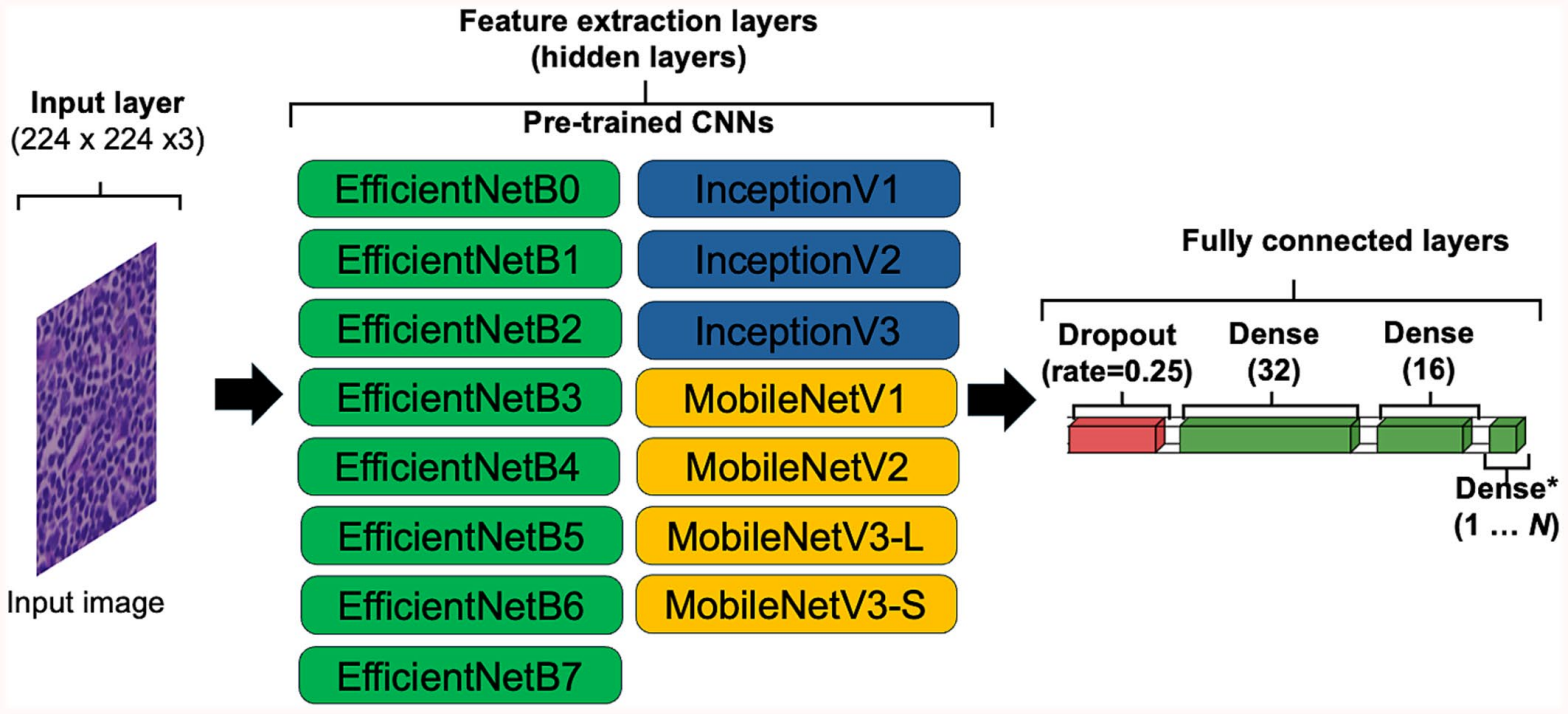
Convolutional neural network (CNN) configuration for transfer learning. To accommodate the default input image sizes of certain pre-trained CNNs, the input image was set to 224 × 224 × 3, ensuring compatibility across all tested architectures. All layers capable of updating weights were set as frozen (i.e., set to non-trainable). The penultimate layer of each CNN was configured to flatten the data, exporting DL feature vectors of varying sizes. A dropout layer with a 10% dropout rate was applied for regularisation, followed by three fully connected dense layers with 32, 16, and 1 neuron, respectively, with the final neuron used for classification.

The use of transfer learning (TL) was explored to determine if pre-trained CNN architectures can outperform models generated using HawksheadNet. Pre-trained models such as EfficientNet (7), Inception (5, 6), and MobileNet (8) were previously demonstrated to be viable CNNs to classify cancer tissues. Various versions of EfficientNet, Inception, and MobileNet were explored (Figure 3). Some of the pre-trained CNNs required specific input size, the input layer was set to 224 pixels in width and height, with RGB channels, resulting in dimensions of 224 × 224 × 3 to maintain comparability with all the downstream layer set to non-trainable. The penultimate dense layer was set as the flatten layer which exports varying sizes of DL feature vectors followed by a dropout layer to randomly drop 25% of the connections which was subsequently fed into three consecutive dense layers, with the final layer set as classifier. The hyperparameters were configured as follows: an initial learning rate of 0.0005 with exponential decay using default settings, a batch size of 512, the AdAM optimiser, a loss function based on binary cross-entropy, and maximum epochs of 100. Early stopping checkpoint was applied with for all pre-trained CNN with patience set to three epochs, which terminates training when validation accuracy does not meaningfully improve.

### 2.9 Whole slide image segmentation overlay

For WSI segmentation, ROIs from individual WSIs from each patient assigned to the testing data set were used. ROIs were tiled into non-overlapping 512 × 512 pixels tiles at 0.22 μm per pixel resolution using QuPath (v0.5.1 ×64) and stain normalised. During the classification, the coordinates and predicted value ŷ of a tile were recorded, where the probability of a tile being classified as cDLBCL or RLH was calculated, with ŷ ∈ [0, 1]. The threshold for the prediction was set such that values closer to 0 are more likely to be RLH, while those nearer to 1 more likely to indicate cDLBCL. A Groovy script was used to overlay predictions on the original WSI in QuPath (v0.5.1 ×64; See Data availability statement).

### 2.10 Computational hardware

Model development and generalisation testing were performed on two machines including: (1) A HPC with AMD Ryzen Threadripper 2,950× 16-Core Processor and (2) Apple MacBook Pro M1 Pro with 16 GB of unified memory. The HPC was used for GAN-based stain normalisation, as well as for training and testing with pre-trained CNNs models. MacBook Pro was employed for stain transfer normalisation and for local training and testing of HawksheadNet models.

### 2.11 Statistical analysis

To assess the training performance, loss and accuracy curves per epoch during training and validation were used. To evaluate the predictive performance of the models, the number of tiles in the testing set was not altered to simulate a real-world setting with imbalanced data. Metrics were used to assess generalisation including accuracy (Equation 1), precision (Equation 2), sensitivity (Equation 3), specificity (Equation 4), F1 score (Equation 5) and area under the receiver operating characteristic (AUROC), which measures the ability of the model to distinguish between classes across all classification thresholds—values closer to 1 indicate better performance (11).

To determine if there is significant difference between AUROC of two models for binary classification, we applied DeLong’s Test. It is a non-parametric method of comparing AUROCs by accounting their variance and covariance (32). To compare the classification performance of two binary models, McNemar’s test was used. McNemar’s test is a non-parametric method that evaluates whether the number of images misclassified by one model but correctly classified by another is significantly different, and vice versa. In this study, we used the term McNemar *b* which counts misclassified images by Model A but correctly classified by the Model B, and McNemar *c* is the sum for correctly classified images by the Model A but misclassified by the Model B (32). For DeLong’s and McNemar tests, we calculated the AUROCs, McNemar b, and McNemar c by testing the models on their respective stain-normalised test images, rather than using only a single dataset. DeLong’s test was implemented using code from https://github.com/yandexdataschool/roc_comparison (Accessed June 17, 2025), while McNemar’s test was calculated using the mcnemar() function from the Python package statsmodels (v0.14.4). The significance threshold was set at *α* = 0.05.

## 3 Equations

### 3.1 Equation 1

$$\text{Accuracy}=\frac{\text{True Positives}+\text{True Negatives}}{\begin{array}{l} \text{True Positives}+\text{True Negatives}+\\ \text{False Positives}+\text{False Negatives} \end{array}}$$

### 3.2 Equation 2

$$\text{Precision}=\frac{\text{True Positives}}{\text{True Postives}+\text{False Positive}}$$

### 3.3 Equation 3

$$\text{Sensitivity}=\frac{\text{True Positives}}{\text{True Positives}+\text{False Negatives}}$$

### 3.4 Equation 4

$$\text{Specificity}=\frac{\text{True Negatives}}{\text{True Negatives}+\text{False Positives}}$$

### 3.5 Equation 5

$$F1\;\text{score}=\frac{2\times \text{Precision}\times \text{Sensitivity}}{\text{Precision}+\text{Sensitivity}}$$

## 4 Results

### 4.1 Patient cohort

**Figure 4:**
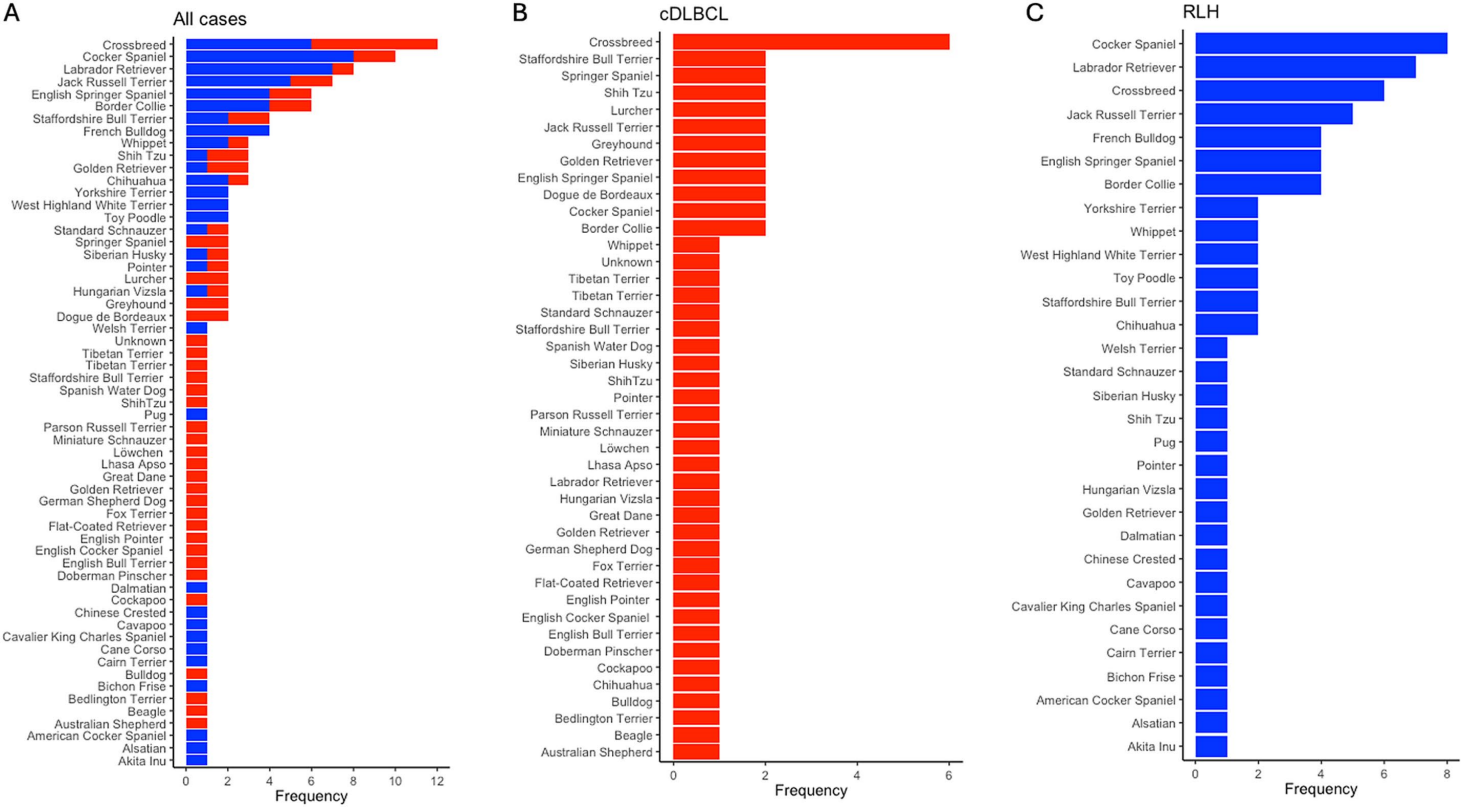
Breed frequency in cDLBCL and RLH cohorts post-filtering. Bar plots showing the frequency of unique breeds post-filtering of images and patients across **(A)** all samples, **(B)** in canine diffuse large B-cell lymphoma (cDLBCL) cases, and in **(C)** reactive lymphoid hyperplasia (RLH) samples. The x-axis indicates the occurrence per breed, while the y-axis lists the breeds from most (top) to least (bottom) represented.

**Figure 5:**
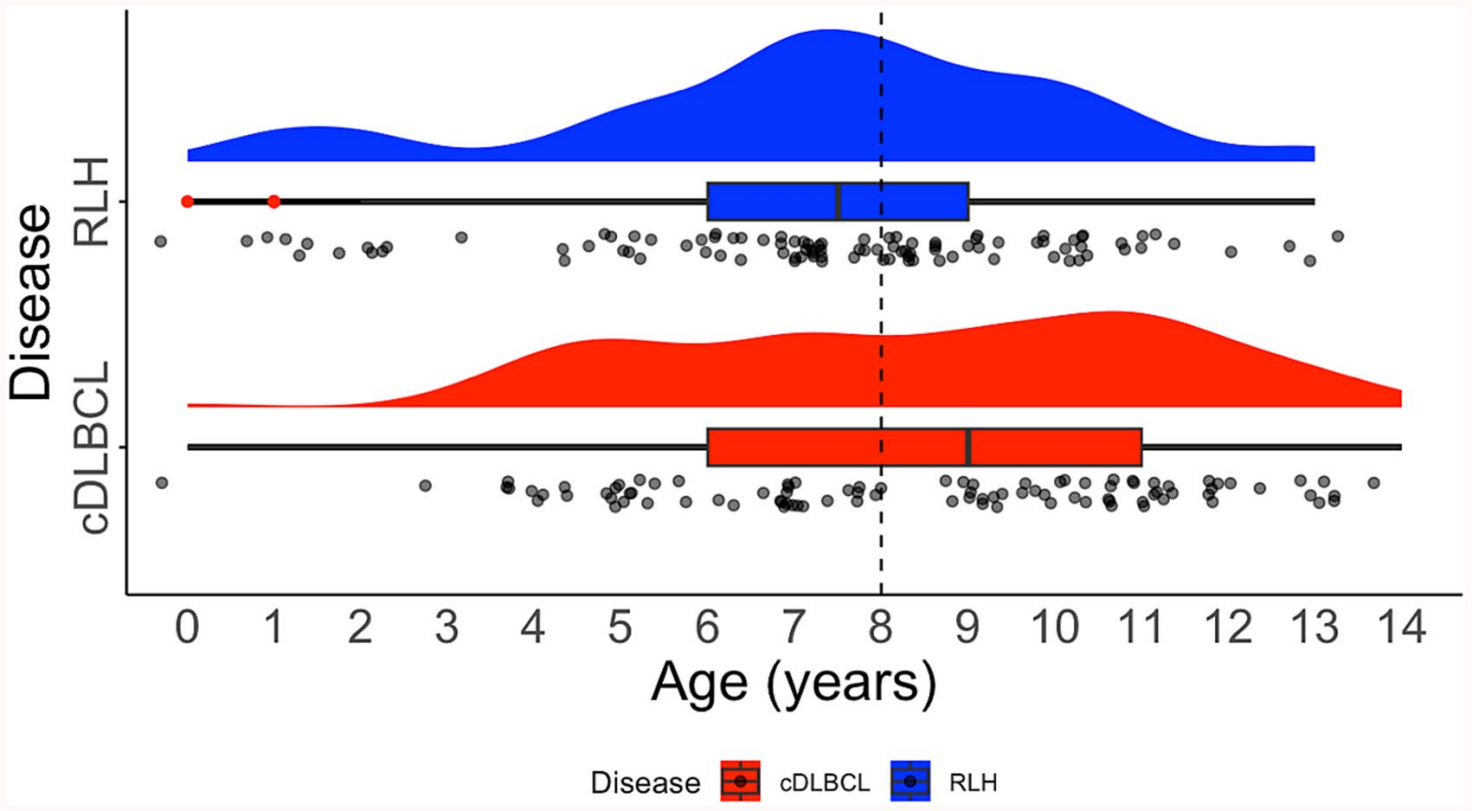
Distribution of age on diagnosis. Raincloud plot showing the age distribution of patients with canine diffuse large B-cell lymphoma (cDLBCL; red) and reactive lymphoid hyperplasia (RLH; blue). Dashed black line indicates the median age for all cases. The raincloud plot integrates half of a violin plot (“cloud”), a boxplot, and jittered raw data points (“rain”). Red dots within the boxplot whiskers denote outliers.

For the entire cohort of 127 samples, there were 67 unique breeds, with the top three being Crossbreed (*N* = 10, 7.87%), Cocker Spaniel (*N* = 8, 6.30%), and Labrador Retriever (*N* = 8, 6.30%). In the cDLBCL group, there were 45 unique breeds, with the most represented being Crossbreed (*N* = 6, 6.78%), followed by 11 breeds tied with two samples each (*N* = 2, 3.39%). In the RLH group, there were 31 unique breeds, with the most represented being Cocker Spaniel (*N* = 8, 11.76%), Labrador Retriever (*N* = 7, 10.29%), and Crossbreed (*N* = 6, 8.83%; Figure 4). The median age at diagnosis for the entire cohort was 8 years (range 1–14), with a median of 9 years (range 3–14) for cDLBCL and 8 years (range 1–13) for RLH. One patient from each class had an undisclosed birth date and age and therefore were excluded from the age ranges (Figure 5).

### 4.2 Tiles for deep learning training

A total of 244,670 and 117,018 images from cDLBCL and RLH cohorts were extracted, respectively, with an overall total of 361,688 pooled tiles. The number of tiles extracted per case had a median of 1,584 (range 29–16,234) for the entire cohort. After limiting the number of tiles per patient based on the median, and ensuring splitting occurred at the patient level, the dataset was divided as follows: 110,603 images for training (DLBCL = 59,395; RLH = 51,208), 28,689 for validation (DLBCL = 16,914; RLH = 11,775), and 16,541 for testing (DLBCL = 7,730; RLH = 8,811).

### 4.3 Stain normalisation speed

The speed of the stain normalisation algorithm depends on the complexity of the calculations and the size of the source image. To determine the processing speeds of each stain normaliser, the average number of processed images/s was calculated. Macenko, Reinhard, Ruifrok, Vahadane, StainNet, and StainGAN achieved average normalisation speeds of 11.58, 109.55, 1.70, 19.31, 48.55, and 0.55 images/s, respectively (Figure 1).

### 4.4 Benchmarking for different stain normalisation protocols using HawksheadNet

**Figure 6:**
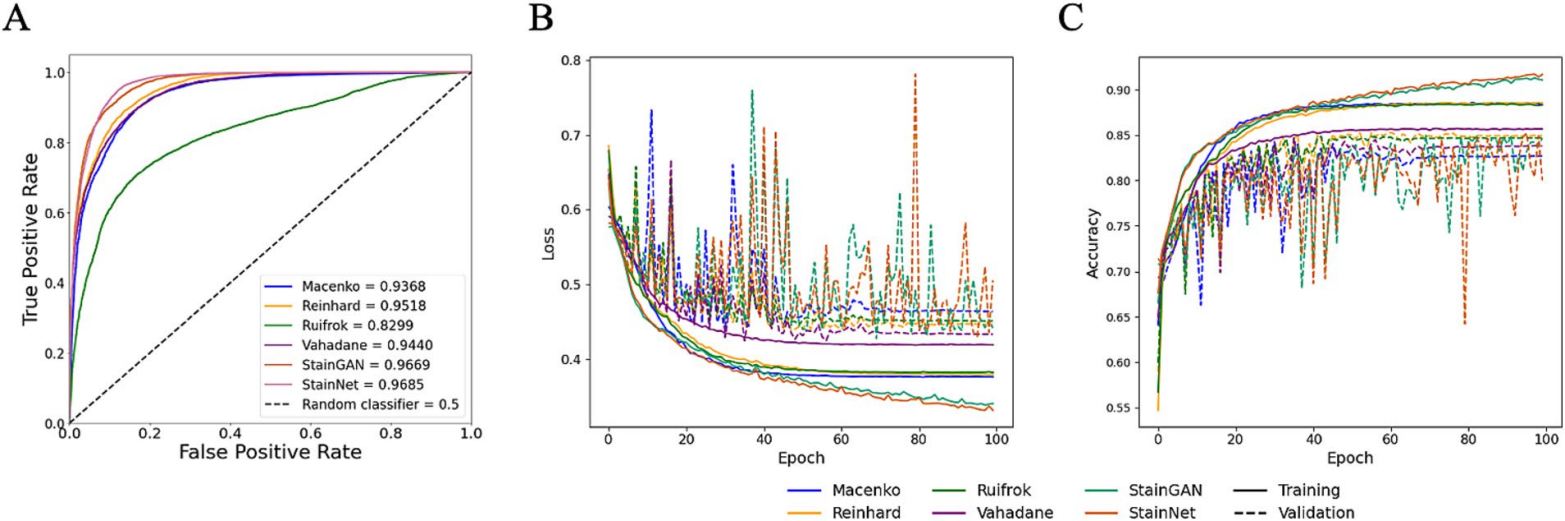
Performance of HawksheadNet trained on different stain normalisers. Overall performance of HawksheadNet using stain transfer-based and generative adversarial network (GAN)-based stain normalisers. Training and testing performances are shown from left to right: **(A)** overall predictive performance was assessed used area under the receiver operating characteristic (AUROC) curve scores, **(B)** training loss (solid line) versus validation loss (dashed line), and **(C)** training accuracy (solid line) versus validation accuracy (dashed line).

While HE-staining of slides in this study was standardised using automatic processes, storage environment (and particularly light exposure) and elapsed time since post-slide preparation (i.e., slide age) are two primary factors that contribute towards HE-stain fading (33, 34). To assess whether different stain normalisation protocols impact DL training performance and downstream generalisation, models were trained on different stain normalisers based on stain transfer and CNN. Models were trained using HawksheadNet to maintain comparability. All models reached a learning plateau at ~80 epochs, except StainGAN and StainNet (Figures 6B,C). The predictive performance was assessed using the test set which was, respectively, stain-normalised (i.e., the model trained on Macenko-adjusted images tested on Macenko-adjusted testing dataset). Models achieved AUROC values between 0.8299 and 0.9685 (Figure 6A). Among stain transfer-based normalisation methods, Reinhard showed the best performance (AUROC = 0.9518), while StainNet demonstrated best performance for GAN-based normalisers (AUROC = 0.9685; Figure 6A). Additional metrics including accuracy, precision, sensitivity (also referred to as recall), specificity, and F1 score were calculated to further evaluate the predictive performance of each model. Reinhard normalisation achieved the highest accuracy of 87.66%, precision of 86.31%, and F1 score of 0.887 among the stain transfer protocols. For GAN-based normalisation, StainGAN achieved superior accuracy of 89.40%, precision of 90.25%, specificity 87.48%, and slightly higher F1 score of 0.9101 compared to StainNet, which only outperformed StainGAN in sensitivity with 98.91% (Table 1).

**Table 1:** Model prediction performance metrics.

Normaliser	CNN	Optimisation	Accuracy	Precision	Sensitivity	Specficity	F1 score
Original hyperparameters							
Macenko	HawksheadNet	None	86.62%	85.44%	90.25%	82.47%	0.8778
Reinhard	HawksheadNet	None	87.66%	86.31%	91.32%	83.49%	0.8874
Ruifrok	HawksheadNet	None	58.30%	56.15%	99.10%	11.79%	0.7169
Vahadane	HawksheadNet	None	86.70%	85.89%	89.79%	83.18%	0.8779
StainGAN	HawksheadNet	None	89.40%	90.25%	92.68%	87.48%	0.9101
StainNet	HawksheadNet	None	88.97%	83.45%	98.91%	77.63%	0.9052
Optimisations							
Reinhard	HawksheadNet	Balanced classes	88.65%	87.45%	91.89%	84.97%	0.8961
	HawksheadNet	Balanced classes and fine-tuning	86.50%	82.80%	94.22%	77.70%	0.8815
	HawksheadNet	Balanced class and regularisers	83.70%	77.27%	98.33%	67.02%	0.8654
StainNet	HawksheadNet	Balanced classes	88.88%	94.19%	84.32%	94.08%	0.8898
	HawksheadNet	Balanced classes and fine-tuning	91.59%	91.88%	92.37%	90.70%	0.9213
	HawksheadNet	Balanced class and regularisers	88.48%	85.20%	94.85%	81.22%	0.8976
Transfer learning							
Reinhard	EffcientNetB0	None	87.15%	87.81%	88.11%	86.05%	0.8796
	EffcientNetB1	None	87.53%	84.23%	94.22%	79.90%	0.8895
	EffcientNetB2	None	88.81%	85.69%	94.82%	81.95%	0.9003
	EffcientNetB3	None	87.92%	86.97%	90.94%	84.48%	0.8891
	EffcientNetB4	None	87.15%	84.32%	93.20%	80.25%	0.8854
	EffcientNetB5	None	85.97%	81.23%	95.81%	74.76%	0.8792
	EffcientNetB6	None	86.92%	83.10%	94.70%	78.05%	0.8852
	EffcientNetB7	None	88.98%	86.40%	94.13%	83.12%	0.901
	InceptionV1	None	84.13%	81.94%	90.06%	77.37%	0.8581
	InceptionV2	None	85.96%	85.03%	89.37%	82.07%	0.8715
	InceptionV3	None	85.19%	83.41%	90.14%	79.56%	0.8664
	MobileNetV1	None	86.34%	84.14%	91.61%	80.32%	0.8772
	MobileNetV2	None	87.30%	86.50%	90.24%	83.95%	0.8833
	MobileNetV3-Large	None	86.60%	86.86%	88.19%	84.80%	0.8752
	MobileNetV3-Small	None	88.19%	88.19%	89.86%	86.29%	0.8902
StainNet	EffcientNetB0	None	86.94%	84.61%	92.27%	80.87%	0.8827
	EffcientNetB1	None	86.99%	84.55%	92.48%	80.74%	0.8833
	EffcientNetB2	None	87.43%	87.25%	89.48%	85.10%	0.8835
	EffcientNetB3	None	86.61%	85.13%	90.70%	81.94%	0.8783
	EffcientNetB4	None	86.12%	84.81%	90.07%	81.62%	0.8736
	EffcientNetB5	None	86.12%	83.94%	91.43%	80.06%	0.8753
	EffcientNetB6	None	86.86%	86.00%	89.99%	83.30%	0.8795
	EffcientNetB7	None	87.90%	87.04%	90.80%	84.59%	0.8888
	InceptionV1	None	85.01%	85.85%	86.04%	83.83%	0.8594
	InceptionV2	None	85.76%	84.90%	89.13%	81.93%	0.8696
	InceptionV3	None	84.85%	85.36%	86.37%	83.12%	0.8586
	MobileNetV1	None	84.99%	84.86%	87.44%	82.21%	0.8613
	MobileNetV2	None	87.66%	86.49%	91.06%	83.79%	0.8872
	MobileNetV3-Large	None	86.62%	86.99%	88.05%	84.99%	0.8752
	MobileNetV3-Small	None	87.05%	84.91%	92.04%	81.36%	0.8833

### 4.5 Fine-tuning data properties and hyperparameter improved generalisation

**Figure 7:**
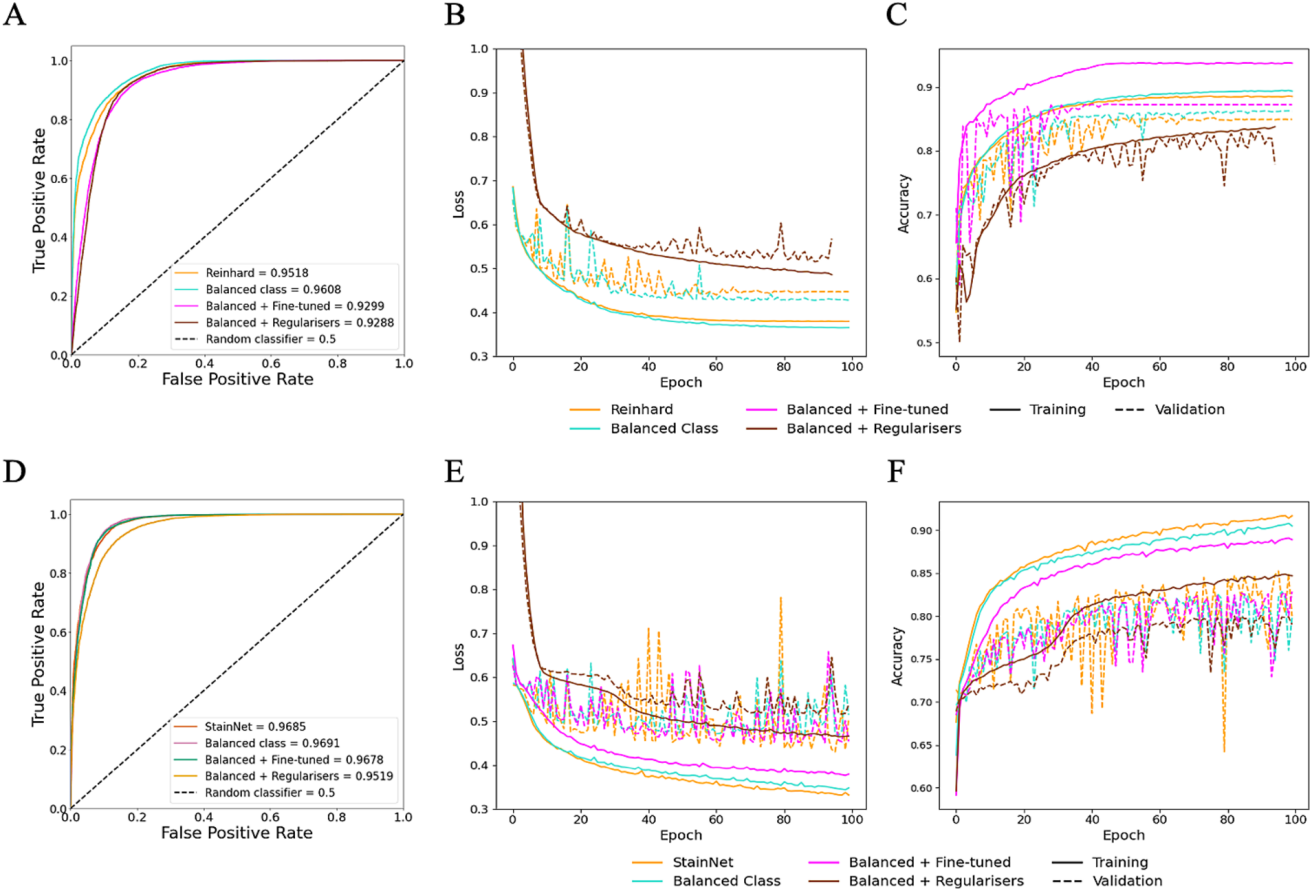
Optimisation of HawksheadNet with the best stain normaliser from stain transfer-based (Reinhard) and generative adversarial network (GAN)-based (StainNet) protocols. Optimisation performance during training and testing for Reinhard and StainNet using HawksheadNet. Metrics are shown for Reinhard in panels **A–C**: **(A)** overall predictive performance assessed using area under the receiver operating characteristic (AUROC) curve scores, **(B)** loss curves, and **(C)** accuracy curves. Corresponding metrics for StainNet are shown in panels **D–F**: **(D)** AUROC curve scores, **(E)** loss curves, and **(F)** accuracy curves. In the loss and accuracy plots, training data are represented with solid lines, and validation data with dashed lines.

To evaluate the impact of dataset properties, such as balance class distribution, and to assess whether hyperparameter fine-tuning can improve generalisation, class balancing and fine-tuning were explored on HawksheadNet models trained using Reinhard-and StainNet-normalised images, which initially demonstrated the highest AUROC scores. Using a normalised test set, the model trained on Reinhard-corrected images showed an increase in AUROC from 0.9518 to 0.9608 when the number of images per class were balanced, however, fine-tuning and the application of L1 and L2 regularisers reduced the AUROC to 0.9299 and 0.9288, respectively (Figures 7A–C). Similarly, the AUROC for the model trained on StainNet-normalised images achieved slight improvement, increasing to 0.9691 for the balanced class and 0.9678 with fine-tuning, while L1 and L2 regularisation reduced it to 0.9619 (Figures 7D–F). Notably, using the fine-tuned parameters resulted in a notable increase in F1 score from 0.9052 to 0.9213 (Table 1).

### 4.6 HawksheadNet outperformed established pre-trained CNNs

**Figure 8:**
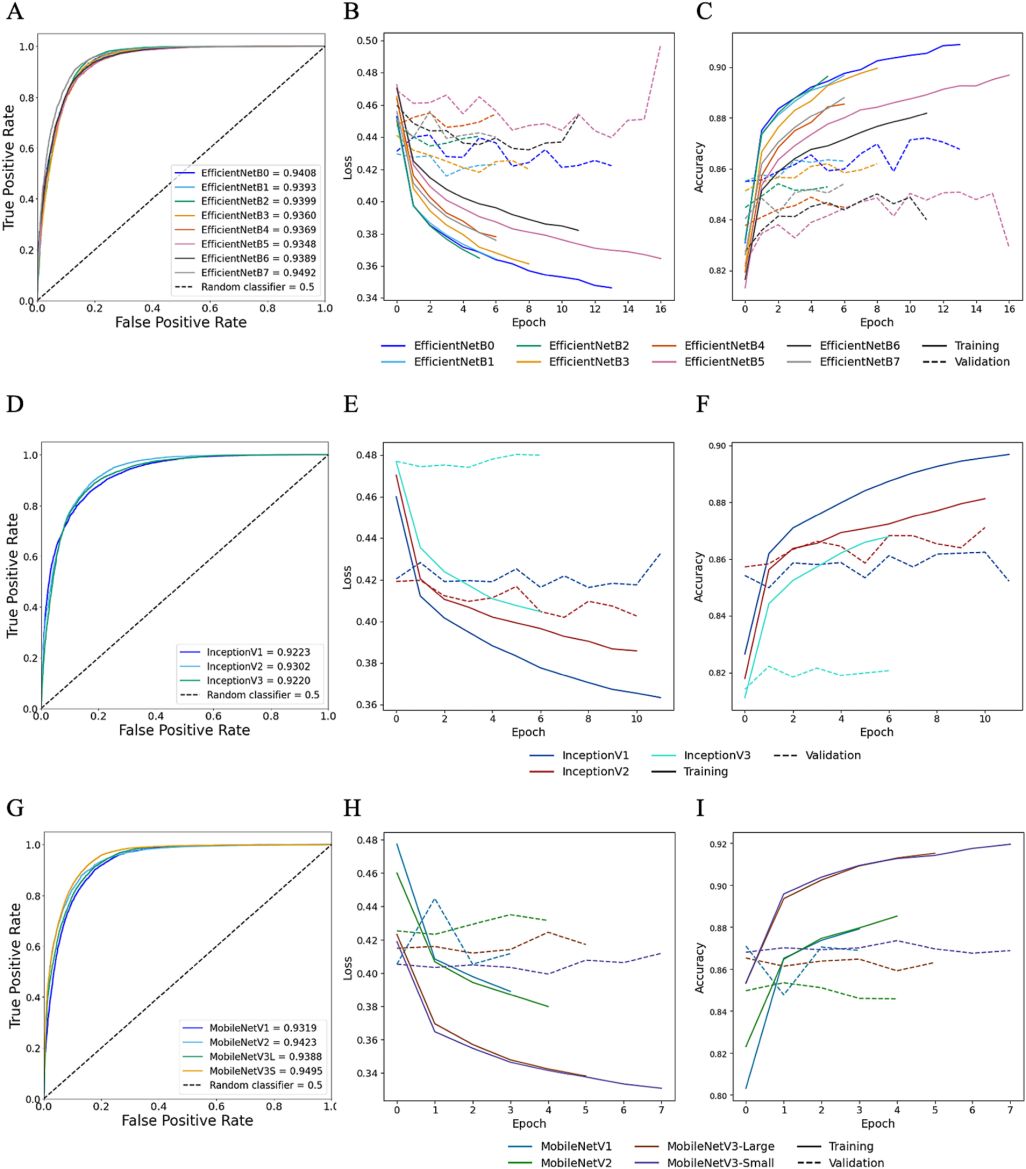
Transfer learning performance using Reinhard normaliser. Training and testing performances for EfficientNet (top row), Inception (middle row), and MobileNet (bottom row) using Reinhard-normalised images. Metrics for EfficientNet are shown in panels **A–C**: **(A)** overall predictive performance assessed using area under the receiver operating characteristic (AUROC) curve scores, **(B)** loss curves, and **(C)** accuracy curves. Metrics for Inception are shown in panels **D–F**: **(D)** AUROC curve scores, **(E)** loss curves, and **(F)** accuracy curves. Metrics for MobileNet are shown in panels **G–I**: **(G)** AUROC curve scores, **(H)** loss curves, and **(I)** accuracy curves. In the loss and accuracy plots, training data are represented with solid lines, and validation data with dashed lines.

TL was employed to determine whether HawksheadNet can achieve comparable generalisation performance to deeper and wider pre-trained CNNs previously used in morphological studies such as EfficientNet (7), Inception (5, 6), and MobileNet (8). Reinhard and StainNet normaliser were employed for the TL model training with balanced classes. For pre-trained CNNs trained on Reinhard-normalised images, the AUROC values ranged from 0.9348 to 0.9492 for EfficientNet (Figures 8A–C), 0.9220 to 0.9302 for Inception (Figures 8D–F), and 0.9319 to 0.9495 for MobileNet (Figures 8G–I). The overall poorest-performing architecture was Inception, with InceptionV3 demonstrating the lowest predictive performance, achieving an AUROC of 0.9220 with F1 score of 0.8664 (Table 1). The mean F1 scores for EfficientNet, Inception and MobileNet architectures were 0.8887, 0.8653, and 0.8814, respectively. MobileNetV3-Small and EfficientNetB7 achieved the highest AUROCs of 0.9495 and 0.9492, respectively (Figure 8). While MobileNetV3-Small had a negligibly higher AUROC, EfficientNetB7 achieved a notably better F1 score of 0.901 compared to 0.875 for MobileNetV3-Small (Table 1).

**Figure 9:**
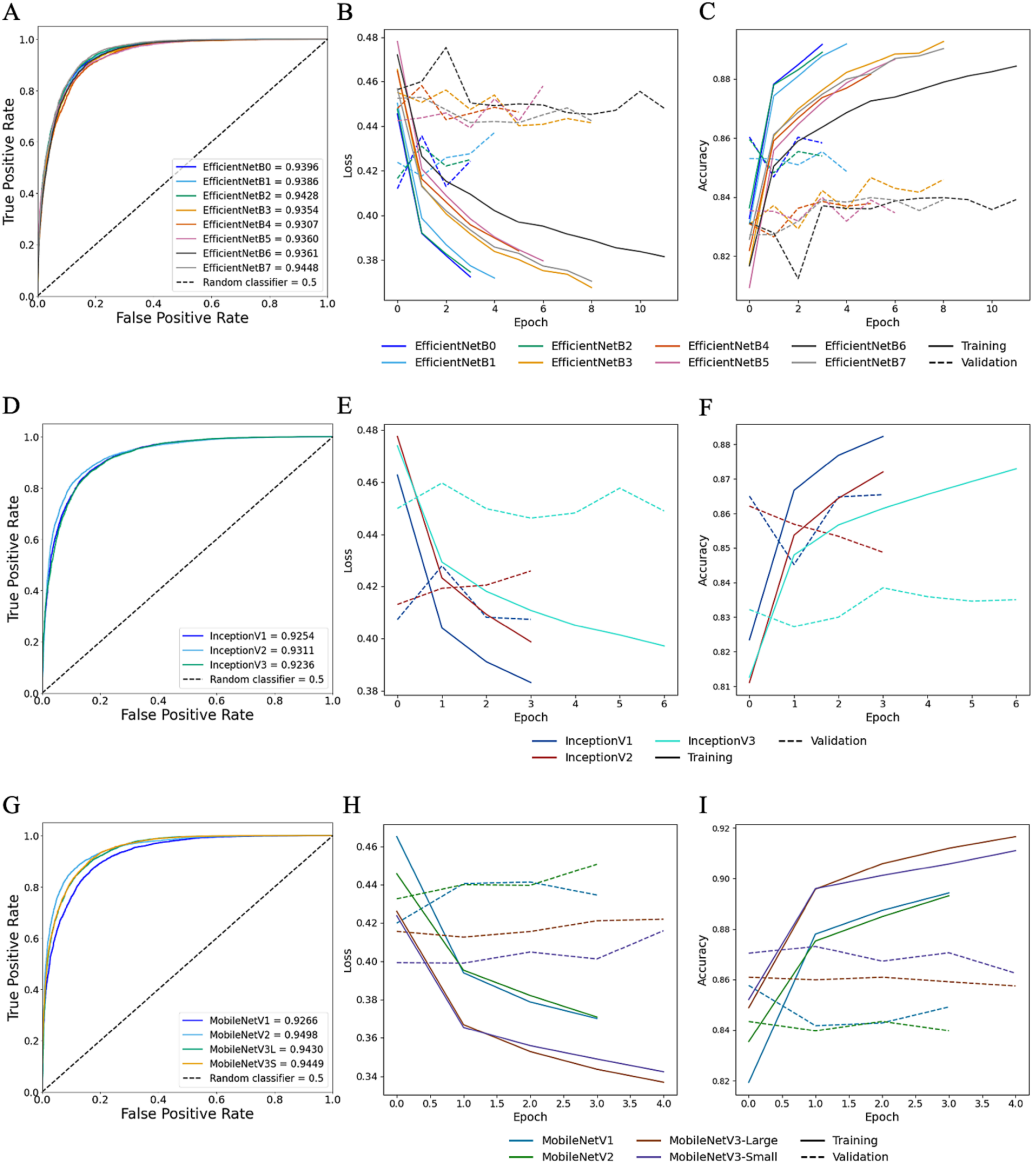
Transfer learning performance using StainNet normaliser. Training and testing performances for EfficientNet (top row), Inception (middle row), and MobileNet (bottom row) using StainNet-normalised images. Metrics for EfficientNet are shown in panels **A–C**: **(A)** overall predictive performance assessed using area under the receiver operating characteristic (AUROC) curve scores, **(B)** loss curves, and **(C)** accuracy curves. Metrics for Inception are shown in panels **D–F**: **(D)** AUROC curve scores, **(E)** loss curves, and **(F)** accuracy curves. Metrics for MobileNet are shown in panels **G–I**: **(G)** AUROC curve scores, **(H)** loss curves, and **(I)** accuracy curves. In the loss and accuracy plots, training data are represented with solid lines, and validation data with dashed lines.

Moreover, for TL trained StainNet-adjusted images the AUROC values ranged from 0.9307 to 0.9448 for EfficientNet, 0.9236 to 0.9311 for Inception, and 0.9266 to 0.9498 for MobileNet (Figure 9). Similar to TL with Reinhard, the worst-preforming CNN in StainNet-normalised images was Inception, with InceptionV3 demonstrating the lowest predictive performance and achieving an AUROC of 0.9236. The mean F1 scores for EfficientNet, Inception and MobileNet architectures were 0.8806, 0.8625, and 0.8767 (Table 1). The best overall CNN architecture based on AUROC was MobileNetV2 with AUROC of 0.9498 and F1 score of 0.8872, while EfficientNetB7 with AUROC of 0.9448 and F1 score of 0.8888 was slightly better when considering the F1 score (Figure 9; Table 1).

The model size generated by EfficientNetB7 was 258.3 MB, with an approximate prediction rate of ~67.45 images/s, compared to MobileNetV2, which resulted in a 9.8 MB model with a rate of ~449.58 images/s (Table 2). Due to the more streamlined and relatively lightweight architecture of MobileNetV2 compared to EfficientNetB7, it was deemed the better TL model for StainNet.

**Table 2:** Different convolutional neural network architectures.

CNNs	Parameters (feature extractor layers)	Final model size (mb)	Images processed per second	Source
HawksheadNet	20,256	0.32–13.5	~6114.18	In-house CNN
EfficientNetB0	4,049,564	17.0	~ 393.57	https://www.kaggle.com/models/tensorflow/efficientnet/TensorFlow2/b0-feature-vector/1
EfficientNetB1	6,575,232	27.2	~300.04	https://www.kaggle.com/models/tensorflow/efficientnet/TensorFlow2/b1-feature-vector/1
EfficientNetB2	7,768,562	32	~260.03	https://www.kaggle.com/models/tensorflow/efficientnet/TensorFlow2/b2-feature-vector/1
EfficientNetB3	10,783,528	44.2	~207.55	https://www.kaggle.com/models/tensorflow/efficientnet/TensorFlow2/b3-feature-vector/1
EfficientNetB4	17,673,816	72–72.4	~162.91	https://www.kaggle.com/models/tensorflow/efficientnet/TensorFlow2/b4-feature-vector/1
EfficientNetB5	28,513,520	115.5	~113.87	https://www.kaggle.com/models/tensorflow/efficientnet/TensorFlow2/b5-feature-vector/1
EfficientNetB6	40,960,136	165.5	~81.37	https://www.kaggle.com/models/tensorflow/efficientnet/TensorFlow2/b6-feature-vector/1
EfficientNetB7	64,097,680	258.3	~67.45	https://www.kaggle.com/models/tensorflow/efficientnet/TensorFlow2/b7-feature-vector/1
InceptionV1	5,607,184	23.1	~572.10	https://www.kaggle.com/models/google/inception-v1/TensorFlow2/feature-vector/2
InceptionV2	10,173,112	41.4	~449.58	https://www.kaggle.com/models/google/inception-v2/TensorFlow2/feature-vector/2
InceptionV3	21,802,784	88.4	~379.57	https://www.kaggle.com/models/google/inception-v3/TensorFlow2/feature-vector/2
MobileNetV1 (1.0 × depth)	3,228,864	13.5	~811.90	https://www.kaggle.com/models/google/mobilenet-v1/TensorFlow2/100-224-feature-vector/2
MobileNetV2 (1.0 × depth)	2,257,984	9.8	~678.21	https://www.kaggle.com/models/google/mobilenet-v2/TensorFlow2/100-224-feature-vector/2
MobileNetV3-Large (1.0 × depth)	1,529,968	17.7	~629.59	https://www.kaggle.com/models/google/mobilenet-v3/TensorFlow2/large-100-224-feature-vector/1
MobileNetV3-Small (1.0 × depth)	4,226,432	6.7	~1251.15	https://www.kaggle.com/models/google/mobilenet-v3/TensorFlow2/small-100-224-feature-vector/1

### 4.7 Model prediction agreement

DeLong’s test revealed no significant difference between the AUROCs of HawksheadNet models trained with different stain normalisers. The AUROCs of the top-performing and optimised HawksheadNet models trained using Reinhard-and StainNet-normalised images were statistically equivalent to those of EfficientNetB7 and MobileNetV2, respectively (Table 3). McNemar’s test suggested significant difference in classification outcomes, indicating that actual prediction per individual images is different. Model trained on Reinhard-adjusted images was able to correctly classify more images than models trained on other stain transfer-based algorithms (Table 3). Ruifrok model appeared to be the worst performing compared to other models. GAN-based normalisers, StainGAN and StainNet both outperformed all stain transfer-based algorithms. When comparing StainGAN and StainNet, StainNet made more classification errors compared to StainGAN (McNemar b = 954 vs. c = 630, *p* < 0.001), which corresponds to a maximum of 5.77% of the total testing set. Among the top-performing models using Reinhard-normalised images, EfficientNetB7 correctly predicted more images than HawksheadNet with balanced classes (McNemar *p* < 0.001; b = 669, c = 1,171), comprising up to 7.08% of the total testing set. For the top-performing StainNet models, fine-tuned HawksheadNet made more correct predictions than MobileNetV2 *b* = 1,359, *c* = 762 accounting for up to 8.22% of the total testing set (Table 3).

**Table 3:** DeLong and McNemar tests for HawksheadNet using stain normalisers and top transfer learning architectures.

CNN 1	CNN 2	Stain normaliser 1	Stain normaliser 2	DeLong *p*-value	McNemar *p*-value	McNemar *b*	McNemar *c*
HawksheadNet	N/A	Macenko	Reinhard	1	0.00469	783	900
HawksheadNet	N/A	Macenko	Ruifrok	1	<0.001	5,227	749
HawksheadNet	N/A	Macenko	Vahadane	1	<0.001	1,044	682
HawksheadNet	N/A	Macenko	StainGAN	1	<0.001	452	1,100
HawksheadNet	N/A	Macenko	StainNet	1	<0.001	725	1,049
HawksheadNet	N/A	Reinhard	Ruifrok	1	<0.001	5,280	685
HawksheadNet	N/A	Reinhard	Vahadane	1	<0.001	1,166	687
HawksheadNet	N/A	Reinhard	StainGAN	1	<0.001	490	1,021
HawksheadNet	N/A	Reinhard	StainNet	1	<0.001	816	1,023
HawksheadNet	N/A	Ruifrok	Vahadane	1	<0.001	422	4,538
HawksheadNet	N/A	Ruifrok	StainGAN	1	<0.001	537	5,663
HawksheadNet	N/A	Ruifrok	StainNet	1	<0.001	91	4,893
HawksheadNet	N/A	Vahadane	StainGAN	1	<0.001	477	1,487
HawksheadNet	N/A	Vahadane	StainNet	1	<0.001	550	1,236
HawksheadNet	N/A	StainGAN	StainNet	1	<0.001	954	630
HawksheadNet (Balanced Class)	EfficientNetB7	Reinhard	N/A	1	<0.001	669	1,171
HawksheadNet (Fine-tuned)	MobileNetV2	StainNet	N/A	1	<0.001	1,359	762

### 4.8 Model prediction provides spatial context via whole slide image segmentation

**Figure 10:**
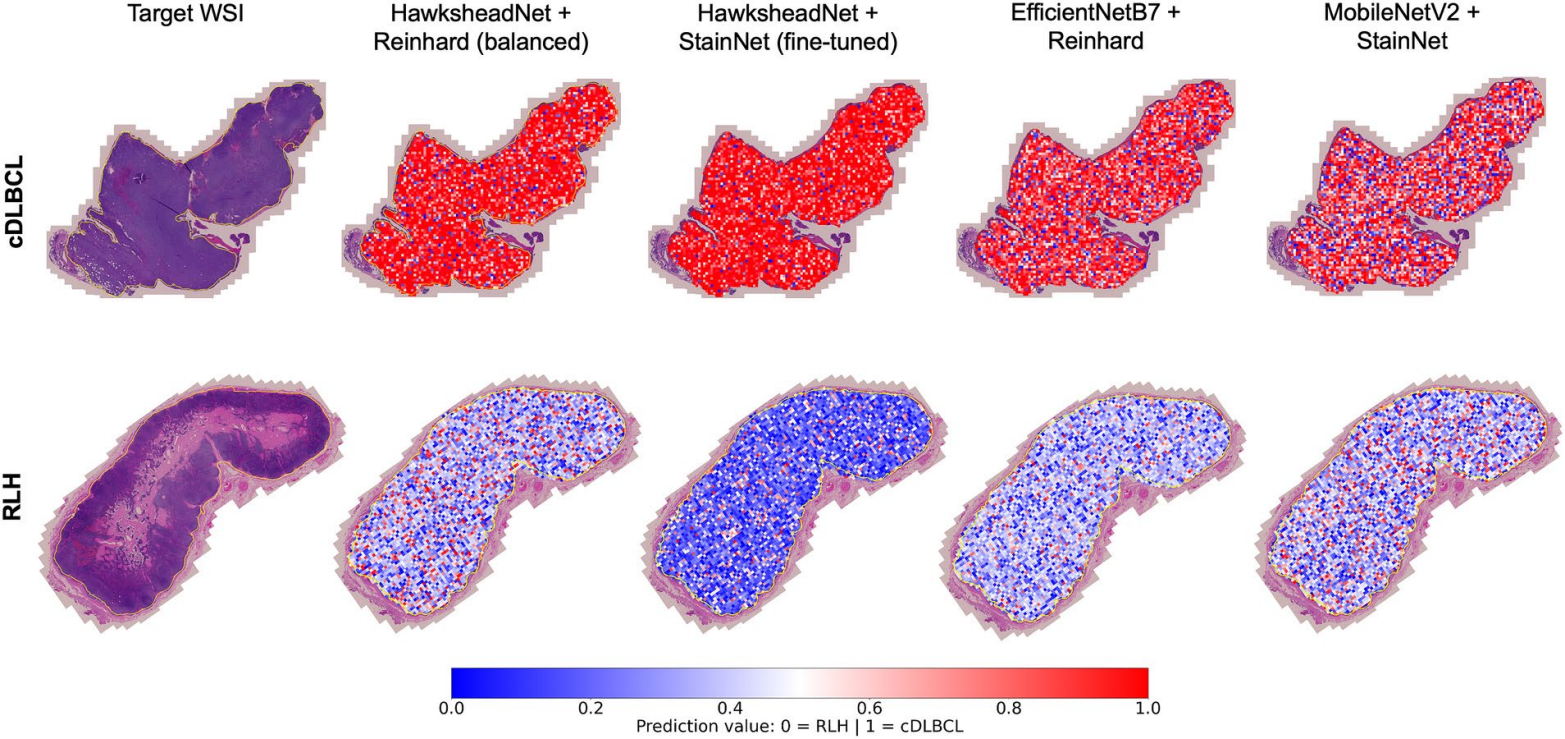
Whole Slide Image (WSI) segmentation. Tile-level predictions were overlaid on the regions of interest within the WSI. Predictions were made using the best-performing models of HawksheadNet and the top networks in transfer learning. The top row shows a true canine diffuse large B-cell lymphoma (cDLBCL) case, while the bottom row shows a true reactive lymphoid hyperplasia (RLH) case. Prediction values closer to 0 (indicated in blue) represent a higher likelihood of non-cancerous or RLH tiles, while values closer to 1 (indicated in red) suggest a higher probability of cDLBCL tiles.

To understand whether the model prediction can provide better spatial context in addition to tile-level prediction, WSI segmentation was conducted by overlaying tile-level predictions on the original HE-stained image. The best performing models generated using HawksheadNet and pre-CNN were used, with their respective stain normalisers from both stain transfer-and GAN-based methods, respectively. In both true cDLBCL and RLH cases, all models appeared to provide predictive visual representation of the WSI status with spatial context (Figure 10). It was notable that some tiles were predicted as more likely to be cDLBCL in RLH WSI, and vice versa. Additionally, all the models seemed to assign higher probability scores when predicting tiles as cDLBCL rather than RLH, as indicated by the darker red shade for cDLBCL and the lighter blue shade for RLH (Figure 10). However, this pattern does not apply to the HawkheadNet + StainNet (fine-tuned) model, which also assigns a higher probability to RLH tiles.

## 5 Discussion

Accurate cDLBCL diagnosis is critical for the timely and guided treatment of patients, directly impacting prognosis and quality of life. While cytological testing may provide a rapid preliminary assessment, confirmatory diagnosis still relies on manual histopathological evaluation, which can be time-consuming and costly. The development of supplementary tools to help veterinary pathologists is urgently needed to help improve diagnostic speed and consistency, improving welfare through better-informed and timely diagnosis.

This study highlights the potential of using CNN models in differentiating cDLBCL from RLH cases. Our results also examined the impact of stain normalisation, various pre-trained CNNs and hyperparameter optimisations on DL computer vision models. Our findings indicate that HawksheadNet is a promising lightweight CNN architecture for binary classification of neoplastic and non-neoplastic lymph nodes. Without fine-tuning, it outperformed different pre-trained CNNs. Its training and predictive performance are further enhanced through appropriate fine-tuning and proper application of stain normalisation.

Stain normalisation of WSIs is crucial for addressing stain inconsistencies prior to DL training, which can introduce image discrepancies and hinder predictive capacity. A plethora of stain normalisation methods are available to standardise histological images for both morphological analysis and DL pre-processing (21, 29). Application of stain normalisation in histological images mitigates these variations, enabling trained models to focus on key features and patterns (35). Madusanka et al. (35), suggested that the impact of stain normalisation is more pronounced in less complex CNN architectures, (i.e., a smaller number of training parameters) as observed in GAN-based normalisers. The number of parameters in the feature extractor layers of pre-trained CNNs varies, with EfficientNet ranging from 4.0 to 64.0 M, Inception from 5.6 to 21.8 M, and MobileNet from approximately 1.5 to 4.2 M, whereas HawksheadNet has only 20,256 parameters. In line with this, the fine-tuned Hawkshead CNN with StainNet performs better than pre-trained CNNs based on AUROC, which also holds true for Reinhard. However, the effect reported by Madusanka et al. (35) does not apply to the pre-trained CNNs we used. This is evident since EfficientNetB7, which has the most parameters (i.e., the largest model), performs best with Reinhard, while MobileNetV2 with the second smallest number of parameters performs best with StainNet, despite their contrasting parameter sizes. Despite the usage of stain normalisation, Hameed et al. (5) demonstrated that the use of non-normalised datasets can result in comparable or better models when trained using stain transfer-based normalisers. While Madusanka et al. (35) and Hameed et al. (5) both focused on breast cancer using GAN-based or stain transfer-based normalisers, we suggest a dynamic and dataset-driven approach in choosing a stain normaliser. This approach would likely involve the retrainable GAN-based architecture designed for stain normalistion like StainNet (29) and StainGAN (28). Future prospects for GAN-based normalisers would ideally require only a small subset of H&E slide patches for robust training, a lightweight architecture requiring less computational resources, fast stain normalisation speed, and user-friendly interfaces. To maintain comparability between stain normaliser, we used ‘stock’ or ‘default’ models from Kang et al. (29). Future research could benefit from retraining StainNet and StainGAN on our own dataset and comparing the outcomes with the stock GAN-based normalisers. Overall, GAN-based normaliser can be adaptively trained independently of reference images, they may offer a more effective option.

In both HawksheadNet with Reinhard and StainNet, class balancing results in a slight improvement in AUROC, while hyperparameter fine-tuning and regularisation negatively affect performance. However, for HawksheadNet with StainNet, although hyperparameter fine-tuning slightly reduced AUROC, it resulted in a significant improvement in the F1 score. These findings highlight that a CNN architecture can respond differently to identical optimisation strategies depending on the stain normaliser used during training. This suggests that stain normalisation influences the sensitivity of CNNs to optimisations. Further investigation is needed to explore whether a broader grid-search for fine-tuning (i.e., more options), including more L1/L2 regularisation values, and longer training epoch per trial could lead to a more robust fine-tuned HawksheadNet CNN.

Pre-trained CNNs like EfficientNet, Inception, and MobileNet present appealing options for morphological analysis. However, to maximise their predictive potential in real-world applications where computational resources are often limited, it is important to factor in performance and computational efficiency when selecting CNN architectures. While our study explores a relatively simple task of distinguishing the most common subtype of canine lymphoma from a reactive lymph node, our findings align with previous studies that pre-trained CNNs are capable of recognising cancer-related morphology. It is worth noting that Inception underperformed compared to all models. While MobileNet and EfficientNet demonstrated comparable results, MobileNet is computationally cheaper and would have more real-world applications. However, our data indicates that the fine-tuned HawksheadNet outperforms all pre-trained CNN we investigated. This highlights the importance of prioritising computational-efficiency and overall generalisation performance when selecting CNN architecture for model training. Further testing on different morphological datasets is required to validate its applicability across varying data types and problem domains.

This is the first study on cDLBCL to demonstrate the application of CNN models for WSI segmentation. A limitation of tile-level classification is its inability to assess larger regions of WSIs. Our results show that model predictions can inform ROI-wide segmentation by superimposing predicted values per patch onto the original HE-stained slide. This approach provides a holistic visual tool to differentiate cDLBCL from RLH, enabling a more comprehensive analysis of the lymph node. WSI segmentation is more pronounced in true cDLBCL cases, displaying a higher concentration of ‘red’ tiles. On the contrary, while RLH cases still exhibit distinguishable segmentation compared to cDLBCL, they contain a greater number of tiles with reduced ‘blue’ intensity, compared to the Hawkshead model with StainNet. While the morphological segmentation achieved by each model varies in the prediction values, the overall visualisation remains consistent with the true diagnosis. Moreover, this highlights the potential to infer additional information from the WSI segmentation, such as immunological hot spots or ‘hot’ tumours. A study by Song et al. (36) on the hDLBCL tumour microenvironment (TME) demonstrated that patients with a ‘hot’ tumour, characterised by a higher infiltration of T-cells and cytokines, experience an increased inflammatory response that helps combat cancerous cells, leading to a better prognosis. In contrast, patients with a ‘cold’ tumour, which have low immune cell infiltration, are associated with immune evasion and a poorer prognosis. Although our current models are not trained to distinguish tumour ‘hotness’ or detect hot spots, they provide rough estimations of regions that are more likely to be cancerous. Future studies could explore inferring ‘hot’ versus ‘cold’ tumour characteristics in WSI-level context rather than tile-level might offer a more comprehensive understanding into tumour heterogeneity, tumour microenvironment patterns and immune responses in canine cancers. This approach could also be extended to infer other ‘-omics’ information, as demonstrated in human studies involving genomic mutations (23, 37) and aberrant genetic expressions (38), which could help in patient stratification and potentially lead to better-informed targeted interventions. This could lead to the development of WSI segmentation models capable of inferring spatial ‘-omics’ data (e.g., transcriptomics and proteomics) directly from HE-stained slides, which could be particularly useful in research settings. Overall, this opens new avenues for using and training CNN models to extract additional prognostic information from HE-stained slides and its application in WSI segmentation context.

We acknowledge that the number of patients which underwent additional IHC and PARR testing does not represent the majority of the cohort in this study. While morphology is reliable in diagnosis of large cell lymphoma and establishing a presumptive diagnosis of cDLBCL, with this lymphoma subtype representing the most commonly diagnosed subtype in dogs, in a minority of cases large cell lymphomas may be later shown to be of T-cell rather than B-cell origin where IHC is possible. Future studies would therefore benefit from a more consistent and complete inclusion of IHC and PARR testing across the entire cohort. However, achieving this is particularly challenging in large-scale retrospective clinical studies like ours, where original FFPE tissue is not available for further testing as it is only retained for a clinically relevant limited time scale post-diagnosis. This lack of further diagnostic information is common in clinical cases and may be due to various factors, such as further alternative diagnostics being conducted by the referring clinician, the presumptive diagnosis being considered sufficient when additional chemotherapy is not pursued, or financial limitations preventing owners from seeking further testing.

With regards to the statistics utilised in our study, Rainio et al. (32) previously assessed various tests for DL models, including DeLong’s and McNemar’s test for binary classification models. Typically, DeLong’s and McNemar’s tests are applied to compare different models, often trained using different CNN architectures or classifiers, evaluated on the same testing dataset. However, we employed these tests to compare models based on the same CNN architecture (HawksheadNet) but trained and tested using different stain normalisation methods. This modified approach may not be the most appropriate, however, there is no current consensus on a superior statistical method for this specific scenario. An alternative is to use a non-normalised test set, however, this can negatively impact predictive performance. This underscores the need for robust statistical methods to evaluate deep learning predictions when comparing stain normalisation techniques for a given CNN architecture. DeLong’s test did not detect a significant difference between the performances of all the model, suggesting that the models exhibit comparable predictive performance. Furthermore, while the discordant classifications among the top models used for WSI segmentation were significant, the number of discordant images accounted for less than 10% of the total testing set. Despite this, the WSI segmentation remained largely consistent with the true diagnosis.

Compared to the only previous study on use of CNN for diagnosing canine lymphoma by Hubbard-Perez et al. (10), which only trained on 1,530 images, our study includes a much larger (images = 155,833) dataset for training and testing from a cohort with more diverse breed representation. Hubbard-Perez et al. (10) worked with considerably fewer whole-slide images and tiles, which allowed them to manually review each extracted tile and select only those most clearly corresponding to disease. This approach would be excessively labour-intensive to do on our dataset. Their method may risk excluding images with borderline diagnoses that CNN models must be able to handle in real-world applications. Moreover, unlike our study, which also focuses on WSI segmentation, theirs prioritised tile-level classification. Their dataset also covered multiple lymphoma subtypes, demonstrating potential for subtyping (10). Nevertheless, our dataset remains smaller than those used in the largest CNN studies on human cases (9, 17, 18, 20, 23, 39, 40). Future research should expand the dataset to include a larger cohort and a wider diversity of canine lymphoma immunophenotypes and histological subtypes, particularly those that are challenging to differentiate (e.g., borderline cases). This will improve model robustness and its applicability in veterinary pathology.

In conclusion, our study demonstrates the effectiveness of HawksheadNet, a lightweight CNN architecture, in cDLBCL image classification, particularly in differentiating from RLH using HE-stained slides. The results underscore the significant role of stain normalisation in CNN training as well as the impact of different optimisation techniques to optimise model training. HawksheadNet achieves generalisation performance comparable to well-established pre-trained CNNs, such as EfficientNet, Inception, and MobileNet. Furthermore, our study confirms that WSI segmentation can be performed by overlaying tile-level predictions onto the original WSI, with WSI-level segmentation closely aligning with true diagnoses. Although DL algorithms, including CNNs, have not yet been formally adopted for veterinary pathology diagnostics, ongoing development and training are essential to improve their performance and facilitate future clinical integration. Special attention should be given to creating user-friendly interfaces and improving workflows, especially in underrepresented fields like veterinary science, which lags behind human pathology in adoption of these technologies. Overall, CNN models including those based on inference approaches, demonstrate significant potential for improving cancer patient diagnosis and stratification, and may ultimately guide treatment decisions and prognostication.

## Data Availability

The WSIs used in this study are not publicly available due to data sharing restrictions set by our industry partner, IDEXX Laboratories Ltd. (Wetherby, United Kingdom), which is interested in further using the data for commercial and proprietary purposes. The code used for tile extraction from QuPath (v0.5x), stain normalisation, CNN model training, and WSI segmentation will be made available upon reasonable request to the corresponding author.
