# Supplementary material for: Deep-learning based morphological segmentation of canine diffuse large B-cell lymphoma
Source: Front Vet Sci. 2025 Aug 25;12:1656976. doi: 10.3389/fvets.2025.1656976 (PMC12415696; doi:10.3389/fvets.2025.1656976)
Supplement: Supplementary file 1 [file Data_Sheet_1.docx]

Supplementary Material

# Supplementary Method 1: Hyperparameter fine-tuning via grid search

Hyperparameter fine-tuning is one of the most challenging tasks for maximising CNN efficacy in pattern recognition and classification. Theoretically, there are infinite combinations of tunable factors applicable to CNN architectures. In this study, Keras Tuner was used to perform a grid search to identify the optimal hyperparameters. The following setting was used for the search: validation accuracy was used as the metric to be maximised during fine-tuning, the maximum epoch per hyperparameter combination was set to 20, and early stopping checkpoint was applied with patience threshold of three epochs which terminates the training when validation accuracy does not significantly improve. The entire search process was conducted over 50 epochs. The series of hyperparameters shown in Supplementary Table 1 were searched. Following the grid search, the top hyperparameter combination shown in Supplementary Table 2 was also used for the training.
