# Supplementary material for: Deep-learning based morphological segmentation of canine diffuse large B-cell lymphoma
Source: Front Vet Sci. 2025 Aug 25;12:1656976. doi: 10.3389/fvets.2025.1656976 (PMC12415696; doi:10.3389/fvets.2025.1656976)
Supplement: Supplementary file 2 [file Table_1.docx]

Supplementary Material

# Supplementary Table 1. Grid search options for model fine-tuning

| Convolutional layers | |
| --- | --- |
| Convolutional-2D layer 1 | Input shape = (128, 128, 3)  Minimum filter value = 32  Maximum filter value = 256  Step = 32  Activation = [ReLU, Tanh, LeakyReLU] |
| Maxpooling-2D layer 1 | Pooling size = (3, 3)  Padding = ‘Same’ |
| Convolutional-2D layer 2 | Minimum filter value = 16  Maximum filter value = 128  Step = 16  Activation = [ReLU, Tanh, LeakyReLU] |
| Maxpooling-2D layer 1 | Pooling size = (3, 3)  Padding = ‘Same’ |
| Connected layers | |
| Global average pooling-2D layer | Default |
| Dropout layer | Minimum dropout value = 0  Maximum dropout value = 0.5  Step = 0.1 |
| Flatten layer | Default |
| Dense layer 1 | Minimum units = 128  Maximum units = 1024  Steps = 128  Activation = [Sigmoid, Softmax] |
| Dense layer 2 | Minimum units = 64  Maximum units = 256  Steps = 64  Activation = [Sigmoid, Softmax |
| Dense layer 3 (Output layer) | Units = 1 (for two classes)  Units = 2 to *N* (for three of more classes)  Activation = [Softmax] |
| Learning rate schedulers | |
| Initial learning rate (ILR) | Value = [0.01, 0.001, 0.0001] |
| Exponent decay | Initial learning rate = ILR  Decay steps = 10000  Decay rate = 0.9 |
| Piecewise constant decay | Boundaries = [10000, 20000]  Values = [ILR, ILR * 0.1, ILR * 0.01] |
| Cosine decay | Initial learning rate = ILR  Decay steps = 10000 |
| Optimisers | |
| Types of optimisers tested | Optimiser = [AdaM, SGD, RMSprop] |
| Loss functions | |
| Binary cross-entropy | Label smoothing = 0.1 |
| Binary focal cross-entropy | Label smoothing = 0.1 |
| Mean squared error | Default |
| Mean absolute error | Default |
| Callbacks | |
| Early stopping | Monitor = validation loss  Patience = 3 |
| Model checkpoint | Default |
