# Supplementary material for: Deep-learning based morphological segmentation of canine diffuse large B-cell lymphoma
Source: Front Vet Sci. 2025 Aug 25;12:1656976. doi: 10.3389/fvets.2025.1656976 (PMC12415696; doi:10.3389/fvets.2025.1656976)
Supplement: Supplementary file 3 [file Table_2.docx]

Supplementary Material

Supplementary Table 2: Fine-tuned sequential setting based on Keras Tuner

| Convolutional layers | Reinhard | StainNet |
| --- | --- | --- |
| Convolutional-2D layer 1 | Input shape = (128, 128, 3)  Filter value = 192  Activation = Tanh | Input shape = (128, 128, 3)  Filter value = 224  Activation = relu |
| Maxpooling-2D layer 1 | Pooling size = (3, 3)  Padding = ‘Same’ | Pooling size = (3, 3)  Padding = ‘Same’ |
| Convolutional-2D layer 2 | Filter value = 112  Activation = Tanh | Filter value = 48  Activation = relu |
| Maxpooling-2D layer 1 | Pooling size = (3, 3)  Padding = ‘Same’ | Pooling size = (3, 3)  Padding = ‘Same’ |
| Global average pooling-2D layer | Default | Default |
| Dropout layer | Dropout value = 0.2 | Dropout value = 0.3 |
| Flatten layer | Default |  |
| Dense layer 1 | Units = 384  Activation = Sigmoid | Units = 896  Activation = Sigmoid |
| Dense layer 2 | Units = 192  Activation = Sigmoid | Units = 256  Activation = Sigmoid |
| Dense layer 3 (Output layer) | Units = 1 (for two classes) | Units = 1 (for two classes) |
| Learning rate schedulers | Cosine decay | Piecewise constant decay |
| Optimisers | RMSprop | AdAM |
| Loss | Binary crossentropy | Binary crossentropy |
